# Suppression of ATP-dependent (S)-NAD(P)H-hydrate dehydratase expression inhibits adipocyte differentiation of 3T3-L1 preadipocytes by increasing excessive accumulation of NADHX

**DOI:** 10.1093/jb/mvaf015

**Published:** 2025-03-21

**Authors:** Kazuki Nakajima, Kodai Takahashi, Masako Tanaka, Mina Kawashima, Koshi Machida, Yoichi Nakao, Keiyo Takubo, Nobuhito Goda

**Affiliations:** Department of Life Science and Medical Bioscience, Graduate School of Advanced Science and Engineering, Waseda University, TWIns, 2-2 Wakamatsu-cho, Shinjuku-ku, Tokyo 162-8480, Japan; Department of Life Science and Medical Bioscience, Graduate School of Advanced Science and Engineering, Waseda University, TWIns, 2-2 Wakamatsu-cho, Shinjuku-ku, Tokyo 162-8480, Japan; Department of Biomedical Sciences, School of Biological and Environmental Sciences, Kwansei Gakuin University, 1 Gakuen Uegahara, Sanda, Hyogo, 669-1330, Japan; Department of Life Science and Medical Bioscience, Graduate School of Advanced Science and Engineering, Waseda University, TWIns, 2-2 Wakamatsu-cho, Shinjuku-ku, Tokyo 162-8480, Japan; Department of Chemistry and Biochemistry, Graduate School of Advanced Science and Engineering, Waseda University, 3-4-1 Okubo, Shinjuku-ku, Tokyo 169-8555, Japan; Department of Chemistry and Biochemistry, Graduate School of Advanced Science and Engineering, Waseda University, 3-4-1 Okubo, Shinjuku-ku, Tokyo 169-8555, Japan; Department of Cell Fate Biology and Stem Cell Medicine, Tohoku University Graduate School of Medicine, 2-1 Seiryo-machi, Aoba-ku, Sendai, Miyagi 980-8575, Japan; Department of Stem Cell Biology, National Institute of Global Healthand Medicine, Japan Institute for Health Security, 1-21-1 Toyama, Shinjuku-ku, Tokyo 162-8655, Japan; Department of Life Science and Medical Bioscience, Graduate School of Advanced Science and Engineering, Waseda University, TWIns, 2-2 Wakamatsu-cho, Shinjuku-ku, Tokyo 162-8480, Japan

**Keywords:** adipocyte differentiation, NAD(P)H repair system, NAD(P)HX dehydratase, NADHX, PPARγ

## Abstract

ATP-dependent (S)-NAD(P)H-hydrate dehydratase (NAXD) is a crucial enzyme in the nicotinamide adenine dinucleotide repair system that regenerates NAD(P)H, an essential electron donor in metabolic redox reactions. NAD^+^-related metabolic pathways connect cellular metabolism and the expression of genes responsible for adipogenesis; however, the biological significance of the NAXD-mediated repair pathway remains unclear. Herein, we showed that NAXD is essential for normal adipocyte differentiation of 3T3-L1 murine preadipocytes. Silencing of the *Naxd* gene attenuated differentiation-induced lipid accumulation with excessive accumulation of hydrated NADH (NADHX) without altering NAD^+^ levels. FK866, a specific inhibitor of NAMPT, further reduced lipid accumulation even in *Naxd*-silenced cells with substantial decrease in NAD^+^. Supplementation with nicotinamide mononucleotide, a precursor of NAD^+^, restored NAD^+^ levels comparably in *Naxd*- and *LacZ*-silenced cells treated with FK866, but failed to recover adipocyte differentiation of *Naxd*-silenced cells to the level of *LacZ*-silenced cells. In contrast, exposure of wild-type 3T3-L1 cells to NADHX recapitulated the *Naxd* deficiency-elicited inhibitory effects on adipocyte differentiation with reduced expression of master transcriptional regulators of adipogenesis, *peroxisome proliferator-activated receptor γ* and *CCAAT/enhancer binding protein α*. These results suggest that NAXD supports normal adipogenesis, in part, by inhibiting excessive accumulation of NADHX.

## Graphical Abstract

**Graphical Abstract:**
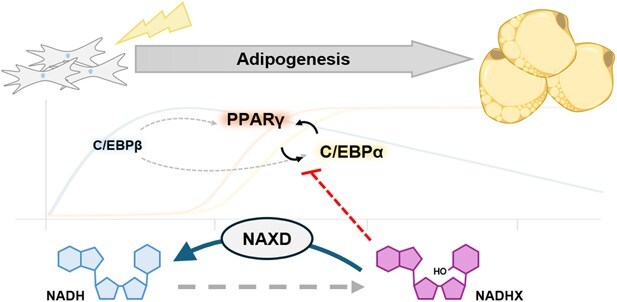

## Abbreviations

C/EBP: CCAAT/enhancer binding protein
ChIP: chromatin immunoprecipitation
Fabp: fatty acid-binding protein
GAPDH: glyceraldehyde-3-phosphate dehydrogenase
LC–MS/MS: liquid chromatography–tandem mass spectrometry
NAD(P)HX: hydrated NAD(P)H
NAXD: ATP-dependent (S)-NAD(P)H-hydrate dehydratase
NMN: nicotinamide mononucleotide
NAMPT: nicotinamide phosphoribosyltransferase
PBS: phosphate-buffered saline
PPAR: peroxisome proliferator-activated receptor
SIRT: sirtuins

Excessive consumption of high-calorie foods and low physical activity cause abnormal accumulation of neutral lipids in the adipose tissue, a condition known as obesity. Obesity is considered a common underlying pathology of various metabolic dysfunctions, such as diabetes, hyperlipidemia, liver steatosis and atherosclerosis. Adipose tissue expansion occurs either by enlarging the size of the adipocytes (hypertrophy) or by increasing the number of adipocytes (adipogenesis). Therefore, controlling adipogenesis with reducing the size of hypertrophic adipocytes is crucial for maintaining healthy adipose tissue and preventing the development of obesity-associated comorbidities.

The regulatory mechanisms of adipocyte differentiation mediated by transcription factors have been extensively explored. Upon adipogenesis, 3T3-L1 preadipocyte cells rapidly and transiently increase the expression of two transcription factors, CCAAT/enhancer-binding protein δ (C/EBPδ) and C/EBPβ, resulting in mitotic clonal expansion *(**1**,*  *2**)*. These changes sequentially induce the expression of the master transcriptional regulators of adipogenesis, peroxisome proliferator-activated receptor γ (PPARγ) and C/EBPα *(**3**,*  *4**)*, whose coordinated induction is a prerequisite for subsequent terminal differentiation into mature adipocyte *(**5**)*. Gene expression and cellular metabolism are closely associated with the production of healthy adipocytes. Among the intermediate metabolites linking these events, NAD^+^ is an essential molecule in adipogenesis, acting not only as an electron acceptor in metabolic redox reactions but also as a substrate for post-translational protein modifications, such as deacetylation and poly-ADP-ribosylation by the NAD^+^-dependent sirtuin (SIRT) family and poly (ADP-ribose) polymerase family, respectively *(**6**,*  *7**)*. NAD^+^ levels increase during adipocyte differentiation, primarily by activating the nicotinamide phosphoribosyltransferase (NAMPT)-mediated salvage pathway. The NAMPT inhibition reduces adipogenesis-induced NAD^+^ biosynthesis and subsequently inhibits adipocyte differentiation. Nicotinamide mononucleotide (NMN), a precursor of NAD^+^, rescues the inhibitory effects of NAMPT inhibition on adipogenesis *(**8**)*. In contrast, adipose tissue in obese mice has decreased levels of NAD^+^, which may impair the production of mature, healthy adipocytes *(**9**)*. These results strongly support the idea that an increase in NAD^+^ levels is a prerequisite for normal adipocyte differentiation and the subsequent formation of mature, healthy adipocytes.

ATP-dependent (*S*)-NAD(P)H-hydrate dehydratase (NAXD), also known as carbohydrate kinase domain-containing protein, is an enzyme that can repair hydrated NAD(P)H (NAD(P)HX) and regenerate NAD(P)H by consuming ATP *(**10**)*. Under stress conditions, such as high temperature or acidic conditions, or by an enzymatic side reaction of glyceraldehyde-3-phosphate dehydrogenase (GAPDH), the nicotinamide ring of NADH is either spontaneously or enzymatically hydrated to form both *S*- and *R*-isomers of NADHX, which are interconverted by NAD(P)HX epimerase, although (*S*)-NADHX serves as a unique substrate for NAXD *(**11**)*. NAD(P)HX no longer functions as an electron donor in redox reactions but can also inhibit the activity of dehydrogenases such as glycerol-3-phosphate dehydrogenase, 3-phosphoglycerate dehydrogenase, glucose 6-phosphate dehydrogenase and 6-phosphogluconate dehydrogenase *in vitro (**12*–*14**)*. Although infection and/or fever appear to trigger NADHX accumulation in patients with mutations in the *Naxd* gene *(**15**)*, little information is available on the physiological and pathological conditions under which NAD(P)HX production and accumulation are enhanced and how NAD(P)HX affects cell/tissue-specific functions. Furthermore, it remains elusive whether the NAD(P)HX repair pathway plays a crucial role in NAD(P)H dynamics and pools.

In this study, we aimed to elucidate the biological significance of NAXD in the regulation of adipocyte differentiation using 3T3-L1 murine preadipocytes and RNA interference-based gene silencing strategy. In addition, we applied a liquid chromatography–tandem mass spectrometry (LC–MS/MS)-based quantification method to determine NAD(H)-related metabolite levels, including NADHX, during adipocyte differentiation. Here, we identified NAXD as a critical regulator in normal adipogenesis presumably by inhibiting excessive accumulation of NADHX.

## Materials and Methods

### Cell culture and adipogenesis induction

3T3-L1 preadipocytes were obtained from the American Type Culture Collection. Cells were cultured in Dulbecco’s modified Eagle medium high glucose (Wako Pure Chemical Industries, Osaka, Japan) containing 10% foetal bovine serum, 100 U/ml penicillin and 100 mg/ml streptomycin at 37°C in 5% CO_2_. PlatE cells, kindly provided by Prof. Toshio Kitamura (The University of Tokyo), were cultured in Dulbecco’s modified Eagle medium low glucose (Wako Pure Chemical Industries) containing 10% foetal bovine serum, 100 U/ml penicillin and 100 mg/ml streptomycin at 37°C in 5% CO_2_. The 3T3-L1 cells were differentiated 2 days after reaching confluence with the induction medium. Adipogenesis induction medium contained 3-isobutyl-1-methylxanthine (Wako Pure Chemical Industries, 0.5 mM), dexamethasone (Dex, Sigma, St. Louis, MO, USA, 1 μM) and insulin (Wako Pure Chemical Industries, 5 μg/ml). Preadipocytes were stimulated by an induction cocktail for 2 days and maintained by insulin (5 μg/ml)-containing medium. In some experiments, FK866 (NAMPT-specific inhibitor, Selleck.co, Houston, USA, 100 nM) was added to the medium 1 h after the addition of differentiation inducer on the differentiation induction day. NMN (NAD^+^ precursor, TCI chemicals, Tokyo, Japan, 0.1 mM) was added to the medium at the same time as the differentiation inducer on the day of differentiation induction. These reagents were added at every culture medium change after the second day of differentiation induction. Rosiglitazone (Sigma, 1 μM) was added to the medium from the second day after differentiation induction. Rosiglitazone was also added at each medium change. NADHX was added to the culture medium for the first 2 days of differentiation.

### Retroviral infection

pMX-U6-puro was a gift from Prof. Toshio Kitamura *(**16**)*. Each shRNA sequence was cloned into a vector. The pMX-U6-sh*LacZ*/*Naxd* vector was transfected into PlatE cells using polyethyleneimine (5 μg, Polysciences, UK) and incubated for 2 days. Virus-containing medium was collected and centrifuged twice (2,330 × *g*, 4°C, 5 min) for debris elimination. After centrifugation, the viral medium was added to the 3T3-L1 dish and incubated overnight. The infected 3T3-L1 cells were selected using 3 μg/ml puromycin (Wako Pure Chemical Industries) for two passages. The shRNA sequences used for gene silencing are listed in Supplemental Table S1.

### Preparation of NADHX

NADHX was prepared by incubating NADH (10 mg) in sodium phosphate solution (400 μl, 0.5 M, pH 7.0) at room temperature for 24 h. This solution was then separated by preparative HPLC (High Performance Liquid Chromatography) using a COSMOSIL 5C18-AR-II column (10 mm I. D. × 250 mm, Nacalai Tesque) with an isocratic solvent system of 8% aqueous MeOH containing 20 mM ammonium acetate at a flow rate of 2.0 mL/min and UV detection at 279 nm to give NADHX. The collected fractions were lyophilized to remove ammonium acetate, yielding NADHX as a dry residue. 

### Oil red O staining

Differentiated 3T3-L1 cells were stained with Oil Red O 6 days after the induction of adipogenesis. For staining, cells were washed twice with phosphate-buffered saline (PBS) and fixed with 4% paraformaldehyde in PBS for 10 min at 37°C. After fixation, cells were washed twice with PBS and rinsed with 60% isopropanol. Cells were stained for 15 min in Oil Red O solution (0.18% (wt/vol) in 60% isopropanol) at 37°C. After removing the dye compound, the cells were rinsed with 60% isopropanol to remove non-specific staining. The staining diagram was photographed after two washes with PBS. The Oil Red O dye was extracted with 100% isopropanol for 1 h. The extracted solution was diluted twice and quantified using a spectrophotometer at 510 nm.

### RNA expression analysis

Total RNA was extracted from the cells using RNAiso Plus (Takara Bio, Shiga, Japan). After quantification of RNA, 1 μg of RNA was used for cDNA synthesis. cDNA was synthesized with ReverTra Ace® qPCR RT Kit (TOYOBO, Osaka, Japan). The cDNA solution was diluted to 100 μl with double distilled water, and 5 μl of the diluted cDNA solution was used for quantitative polymerase chain reaction (qPCR). Finally, the expression level was evaluated using the Step One PlusTM qPCR system (Applied Biosystem, Waltham, USA) and GoTaq® qPCR Master Mix (Promega, Madison, USA). The PCR protocol consisted of 95°C for 10 min followed by 40 cycles of 95°C for 15 s and 60°C for 1 min. The sequences of the primers used for the analysis are shown in Supplemental Table S2. The expression level was normalized to the level of *18S rRNA* as an internal control.

### Nuclear protein extraction

For nuclear protein extraction, nuclei were extracted from cells using a hypotonic buffer (10 mM HEPES-KOH (pH 7.9), 1.5 mM MgCl_2_, 10 mM KCl and protease inhibitor). After 15 min of incubation in a hypotonic buffer for 15 min, 10% NP-40 was added (0.5%), and the suspension was vortexed for 10 s. The nuclear fraction was precipitated by centrifugation (20,400 × *g*, 4°C, 5 min). Extraction buffer (10 mM Tris–HCl (pH 7.4), 100 mM NaCl, 1% TritonX-100, 1 mM EDTA, 1 mM EGTA, 0.1% SDS, 1 mM sodium fluoride, 0.5% sodium deoxycholate and protease inhibitor) was added to the nuclear pellets after centrifugation and sonicated four times for 30 s each. The samples were centrifuged (20,400 × *g*, 4°C, 15 min) to eliminate debris. The amount of cellular protein was evaluated using a BCA assay kit (Wako Pure Chemical Industries), and samples were boiled with 1 × Sample buffer at 95°C, 5 min.

### Western blotting

Protein samples (20 μg) were loaded onto 12.5% polyacrylamide gels and separated through electrophoresis. The proteins were transferred onto polyvinylidene difluoride membranes (Merck Millipore, Darmstadt, Germany). The antibodies used for protein probes are listed in Supplemental Table S3. Chemiluminescent signals were detected using ChemiDoc XRS+ systems (Bio-Rad, CA, USA), and the densitometry of each blot was quantified using Image Lab software (Bio-Rad). Uncropped images of western blots are shown in Supplemental Fig. S1.

### Chromatin immunoprecipitation-PCR

3T3-L1 cells collected on Day 1 after adipogenic stimulation were cross-linked with 1% methanol-free formaldehyde for 10 min at room temperature. The cross-linking reaction was quenched using glycine (0.125 M). After washing twice with PBS, the cross-linked cells were collected in a centrifuge tube using a cell scraper. Collected cell pellets were frozen in liquid N_2_ and thawed rapidly in 37°C water baths. Nuclear extraction buffer (50 mM Tris–HCl (pH 7.5), 140 mM NaCl, 1 mM EDTA, 0.5% NP-40 and protease inhibitor) was added, and the cells were dispersed using a 21G needle (Terumo Corp., Tokyo, Japan). After nuclear collection, 2 ml of chromatin immunoprecipitation (ChIP) dilution buffer (50 mM Tris–HCl (pH 7.5), 140 mM NaCl, 1 mM EDTA, 0.1% SDS, 0.2% TritonX-100, 0.5% NP-40 and protease inhibitor) was added, and nuclear pellets were sonicated. The amount of chromatin protein was evaluated using a bicinchoninic acid assay kit and diluted to the same concentrations. Then, 2 μg of IP antibody (anti-C/EBPβ (H-7): sc-7962) and Dynabeads™ Protein G (Thermo Fisher Scientific, MA, USA) were added to samples and rotated overnight at 4°C. The magnetic beads–antibody complex was washed with a low-salt buffer (20 mM Tris–HCl (pH 8.0), 150 mM NaCl, 2 mM EDTA, 0.1% SDS and 1% Triton X-100), high-salt buffer (20 mM Tris–HCl (pH 8.0), 500 mM NaCl, 2 mM EDTA, 0.1% SDS and 1% Triton X-100), LiCl buffer (10 mM Tris–HCl (pH 8.0), 250 mM LiCl, 1 mM EDTA, 1% NP-40 and 1% sodium deoxycholate) and TE buffer (10 mM Tris–HCl (pH 8.0) and 1 mM EDTA), twice each. Cross-link and protein–antibody binding was removed by incubating with the elution buffer (100 mM NaHCO_3_, 1% SDS and 0.8 mg/ml Protease K) overnight at 65°C. The DNA fragment was purified using Wizard® SV Gel and PCR Clean-Up System (Promega). Immunoprecipitated DNA and 10% input DNA were analysed using a StepOnePlus PCR system (Applied Biosystems). Data are presented as a relative percentage of the input to the control. The sequences of the primers used for the analysis are listed in Supplemental Table S4.

### Measurement of NAD^+^-related metabolites

The metabolome was extracted from differentiated preadipocytes at each time point. Preadipocytes were scraped with methanol containing the internal standard 13C5 adenosine (1 μM), and water was added to be 80% methanol. The diluted cells were sonicated in ice-cold water for 30 s and centrifuged (9,100 × *g*, 4°C, 10 min). After isolating the supernatant, the precipitate was used for protein quantification and normalization. Chloroform was added to the supernatant, centrifuged (20,400 × *g*, 4°C, 15 min) and the upper layer was collected. The supernatant was filtered through a 5-kDa cut filter, freeze-dried and resuspended in 50 mM Ammonium acetate. The resuspended metabolome was quantified using QTRAP 4600 (AB SCIEX, Framingham, USA). Ammonium acetate (50 mM) was used as solvent A, and acetonitrile was used as solvent B. The gradients of solvents A and B are shown in Supplemental Table S5. A Polaris C18-A (Agilent Technologies, CA, USA) was used as an analytical column. The *m/z* values and various settings for mass spectrometry are listed in Supplemental Table S6. The amount of each metabolite was calculated from the standard curve and normalized by the peak area value of 13C5 adenosine as an internal standard. This correction value was again normalized by the amount of precipitated protein. The value was expressed as zero when the measured value was below the detection limit.

### Statistical analysis

**Fig. 1:**
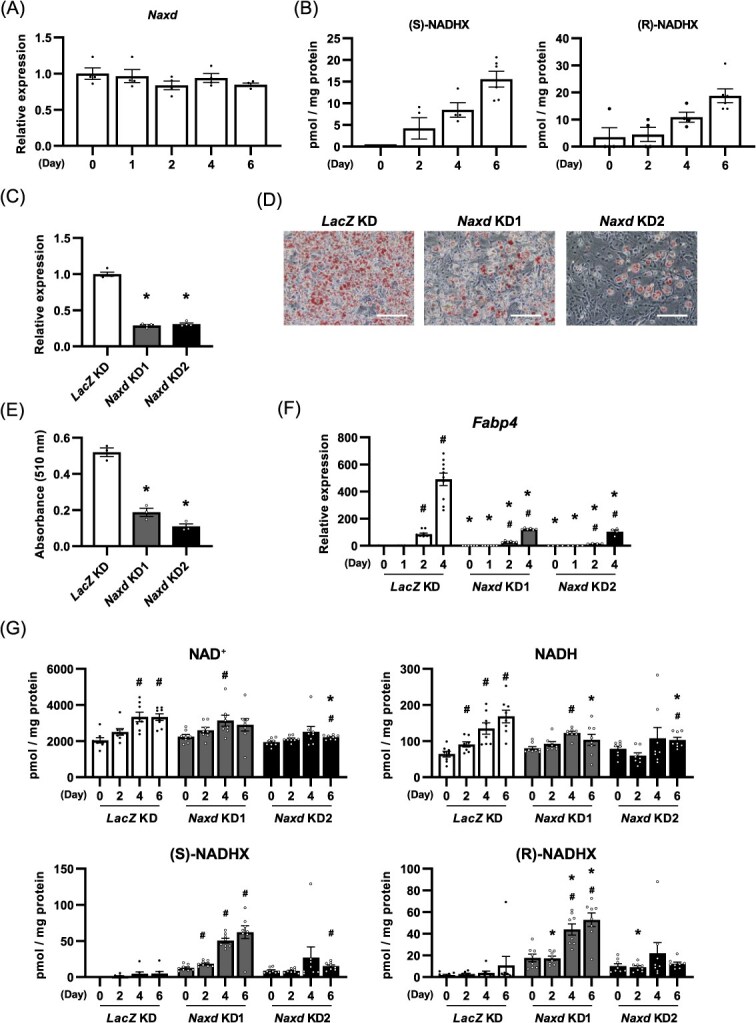
Suppression of *Naxd* expression inhibits adipocyte differentiation with excessive NADHX accumulation. (A) Relative quantification of *Naxd* mRNA in 3T3-L1 cells during adipocyte differentiation. Data represent mean ± SEM; *n* = 4 per group. (B) Quantitative analysis of NADHX in undifferentiated and differentiating 3T3-L1 cells. Data represent mean ± SEM; *n* = 4 (Day 0–4 after adipogenic stimulation) and 6 (Day 6 after adipogenic stimulation) per group. (C) Relative quantification of *Naxd* mRNA in *Naxd* KD. Data represent mean ± SEM; *n* = 4 per group. ^*^P < 0.05 compared to *LacZ* KD. (D), (E) Representative images of Oil red O staining (D) and corresponding quantification (E) of Oil red O of *Naxd* KD on Day 6 after adipogenic stimulation. Scale bar, 200 μm. Data represent mean ± SEM; *n* = 3 per group. ^*^P < 0.05 compared to *LacZ* KD. (F) Relative quantification of *Fabp4* mRNA during adipocyte differentiation of *Naxd* KD. Data represent mean ± SEM; *n* = 10 (*LacZ* KD), 6 (*Naxd* KD1) and 4 (*Naxd* KD2) per group. ^*^P < 0.05 compared to *LacZ* KD on the same day after adipogenesis induction. ^#^*P* < 0.05 compared to Day 0 of the same group. (E) Quantitative analysis of NAD^+^, NADH and NADHX in *Naxd* KD during adipocyte differentiation. Data represent mean ± SEM; *n* = 8 per group. ^*^P < 0.05 compared to *LacZ* KD on the same day after adipogenesis induction. ^#^*P* < 0.05 compared to Day 0 of the same group.

All data shown represent the mean ± SEM. At least three biological replicates were performed in all studies using cell cultures. All data were analysed using Student’s *t*-test, and *P* < 0.05 was considered statistically significant. As the measured value of NADHX in some samples of wild-type (time 0 and 2 day for (S)- and (R)-NADHX in Fig. 1B) and *LacZ* KD (time 0, 2, 4 and 6 day for (S)-NADHX and time 0 day for (R)-NADHX in Fig. 1G) cells was below the detection limit, no statistical analysis was performed for comparison.

## Results

### Suppression of *Naxd* expression inhibits adipocyte differentiation with excessive accumulation of NADHX

The activation of NAD^+^ synthesis through the salvage pathway is important for adipocyte differentiation *(**8**)*. To examine whether NAXD also plays a role in adipocyte differentiation by repairing hydrated NADH to maintain NAD(H) levels, we first determined the changes in *Naxd* expression and the amount of NADHX, a substrate of NAXD, in 3T3-L1 preadipocytes in response to adipogenic induction. The mRNA levels of *Naxd* did not change during adipocyte differentiation (Fig. 1A). Targeted LC–MS/MS analysis revealed that both (*S*)- and (*R*)-NADHX were barely detectable in undifferentiated cells. In contrast, adipogenic stimulation increased the levels of these metabolites in a time-dependent manner (Fig. 1B), indicating differentiation-enhanced production and accumulation of NADHX in 3T3-L1 cells. To investigate the biological importance of NAXD in adipocyte differentiation, we next silenced the expression of *Naxd* gene expression in 3T3-L1 cells and induced adipocyte differentiation. *Naxd* gene was successfully suppressed using retroviral vectors that targeted two different sequences of the gene (Fig. 1C), and knockdown (KD) of the *Naxd* gene markedly inhibited lipid accumulation, as assessed using Oil Red O staining, in response to adipogenic stimuli compared to *LacZ*-silenced cells (*LacZ* KD) (Fig. 1D and E). Consistent with these results, the expression level of *Fabp4*, a marker gene for adipocyte differentiation, significantly decreased on Day 2 in *Naxd*-silenced cells (*Naxd* KD) compared to *LacZ* KD (Fig. 1F). These results suggest that NAXD positively regulates adipocyte differentiation.

We next determined whether silencing the *Naxd* gene might show some effects on NAD(H) levels during adipocyte differentiation. The levels of these metabolites significantly increased after Day 4 of differentiation in *LacZ* KD, whereas both metabolites slightly increased in response to adipogenic stimuli in *Naxd* KD (Fig. 1G). However, the levels of both NAD^+^ and NADH in *Naxd* KD did not differ substantially from those in *LacZ* KD during differentiation (Fig. 1G). In contrast, (*S*)- and (*R*)-NADHX were detected even in undifferentiated *Naxd* KD, but not *LacZ* KD, and these metabolites tended to further increase in response to adipogenic stimuli in *Naxd* KD (Fig. 1G). These results suggest that NAXD constitutively repairs and inhibits NADHX accumulation in both undifferentiated and differentiating cells.

**Fig. 2:**
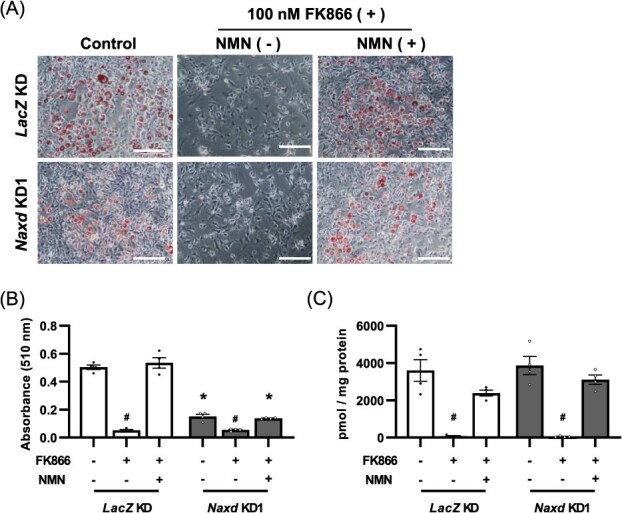
NMN treatment does not rescue inhibition of adipocyte differentiation in *Naxd*-silenced cells. Representative images of Oil red O staining (A) and corresponding quantification of Oil red O (B) of *LacZ* KD and *Naxd* KD1 treated either with FK866 (100 nM) alone or simultaneously with FK866 and NMN (100 μM) on Day 6 after adipogenic stimulation. Scale bar, 200 μm. Data represent mean ± SEM; *n* = 4 per group. *P < 0.05 compared to *LacZ* KD on the same treatment. ^#^*P* < 0.05 compared to the non-treated group on the same cell line. (C) Quantitative analysis of NAD^+^ in *LacZ* KD and *Naxd* KD1 treated either with FK866 (100 nM) alone or simultaneously with FK866 and NMN (100 μM) on Day 4 after adipogenic stimulation. Data represent mean ± SEM; *n* = 4 per group. ^#^*P* < 0.05 compared to the non-treated group on the same cell line.

To examine whether the inhibitory effects of *Naxd* silencing on adipocyte differentiation were attributable to a failure to increase in NAD^+^ levels, we treated cells with FK866, a specific inhibitor of NAMPT, to reduce NAD^+^ levels. As *Naxd* KD2 exhibited a marked inhibition of adipocyte differentiation, which impeded the evaluation of impact of depletion and supplementation of NAD^+^ on adipocyte differentiation, we used only *Naxd* KD1 in this experiment. As expected, FK866 treatment significantly suppressed lipid accumulation with decreased NAD^+^ levels not only in *LacZ* KD but also even in *Naxd* KD1 (Fig. 2A and B), supporting an idea that NAMPT-mediated NAD^+^ salvage is a prerequisite for adipocyte differentiation. Supplementation with NMN, a precursor of NAD^+^, completely abolished effects of FK866 on adipocyte differentiation in *LacZ* KD (Fig. 2A and B), restoring lipid accumulation to the level of untreated *LacZ* KD. In contrast, NMN treatment failed to restore lipid accumulation in FK866-treated *Naxd* KD1 to the level of *LacZ* KD treated with FK866 and NMN (Fig. 2A and B), although the recovery of NAD^+^ levels was comparable between *Naxd* KD1 and *LacZ* KD (Fig. 2C). Collectively, these results suggest that the suppression of *Naxd* expression inhibits adipocyte differentiation without affecting NAD^+^ levels.

### Silencing the *Naxd* gene inhibits differentiation-evoked expression of key transcription factors, *Cebpa* and *Pparg*, in 3T3-L1 cells

**Fig. 3:**
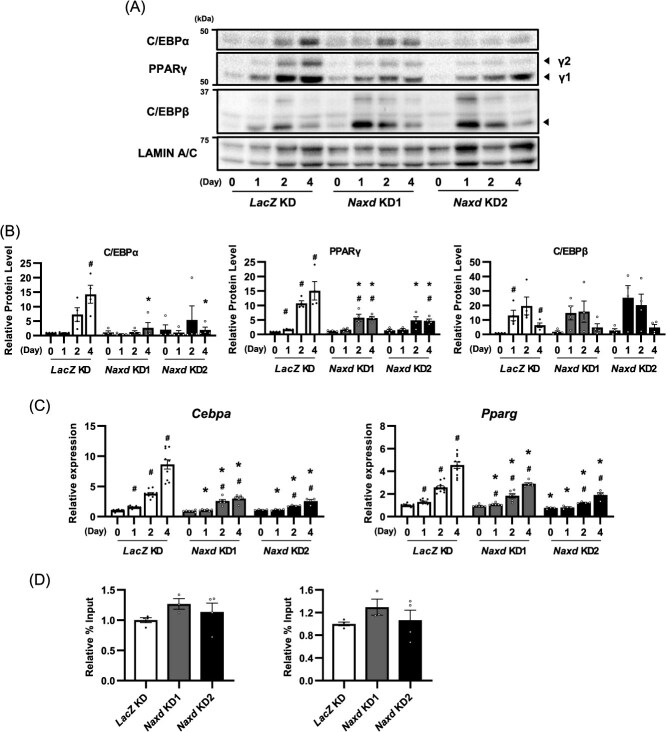
Silencing of the *Naxd* gene reduces differentiation-induced expression of *Pparg* and *Cebpa*. Immunoblots (A) and corresponding quantification (B) of transcription factors involved in adipocyte differentiation in *Naxd* KD after adipogenic stimulation. The expression of LAMIN A/C was used as an internal control. Data represent mean ± SEM; *n* = 4 per group. *P < 0.05 compared to *LacZ* KD on the same day after adipogenesis induction. ^#^*P* < 0.05 compared to Day 0 of the same group. (C) Relative quantification of *Cebpa* and *Pparg* in *Naxd* KD after adipogenic stimulation. Data represent mean ± SEM; *n* = 10 (*LacZ* KD), 6 (*Naxd* KD1) and 4 (*Naxd* KD2) per group. *P < 0.05 compared to *LacZ* KD on the same day after adipogenesis induction. ^#^*P* < 0.05 compared to Day 0 of the same group. (D) ChIP-qPCR analysis of C/EBPβ at the promoter of *Cebpa* and *Pparg* gene in *Naxd* KD 1 day after adipogenic stimulation. Data represent mean ± SEM; *n* = 4 (*LacZ* KD and *Naxd* KD2) and 3 (*Naxd* KD1) per group.

Coordinated expressions of two key adipogenic transcription factors, C/EBPα and PPARγ, are critical for adipocyte differentiation initiation. Adipogenic stimuli markedly increased protein levels of both C/EBPα and PPARγ in the nucleus of LacZ KD on Day 2 of differentiation and thereafter. However, as expected, silencing of *Naxd* significantly suppressed the induction of these proteins compared to that in *LacZ* KD (Fig. 3A and B). Consistent with these results, the mRNA levels of both *Cebpa* and *Pparg* gradually increased after Day 2 of differentiation in *LacZ* KD, whereas these alterations were substantially inhibited by silencing *Naxd* gene (Fig. 3C). Nuclear protein levels of an upstream transcription factor for these key transcription factors in adipocyte differentiation, C/EBPβ, was transiently induced in *LacZ* KD after Day 1 of differentiation, and silencing the *Naxd* gene did not have any impact on the transient increase in C/EBPβ protein levels (Fig. 3A and B). To examine whether the suppressed expression of both *Cebpa* and *Pparg* was caused by a failure of C/EBPβ binding to the promoter regions of *Cebpa* and *Pparg* genes, we performed a ChIP-PCR assay using a specific antibody against C/EBPβ. In response to adipogenic stimuli, C/EBPβ successfully bound to the promoter region of both *Cebpa* and *Pparg* genes in *LacZ* KD after Day 1 of differentiation (Supplemental Fig. S2). Unexpectedly, *Naxd* silencing did not impair the binding of C/EBPβ onto the promoter region of these genes (Fig. 3D), suggesting a dispensable role of C/EBPβ in the suppression of *Cebpa* and *Pparg* gene expressions in *Naxd* KD. These results suggest that NAXD controls adipocyte differentiation by regulating the expression of *Cebpa* and *Pparg*.

### Rosiglitazone, a PPARγ activator, restores adipocyte differentiation in *Naxd* KD cells

**Fig. 4:**
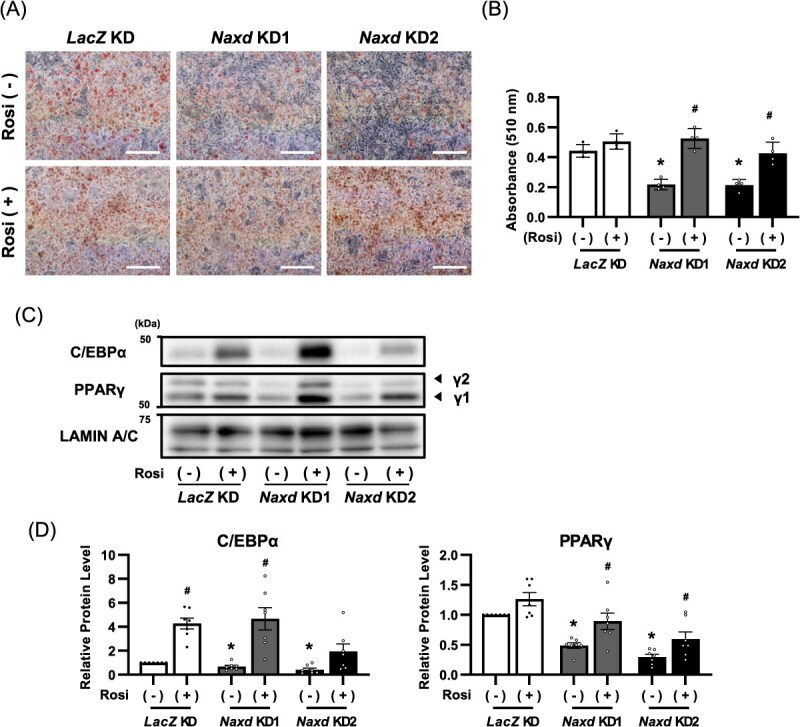
Rosiglitazone restores inhibition of adipocyte differentiation by suppressing *Naxd* expression. Representative images of Oil red O staining (A) and corresponding quantification of Oil red O (B) of *Naxd* KD treated with or without Rosiglitazone (1 μM) on Day 6 after adipogenic stimulation. Scale bar, 200 μm. Data represent mean ± SEM; *n* = 4 per group. ^*^P < 0.05 compared to *LacZ* KD on the same treatment. ^#^*P* < 0.05 compared to the non-treated group on the same cell line. Representative immunoblots (C) and corresponding quantification (D) of C/EBPα and PPARγ of *Naxd* KD treated with or without Rosiglitazone (1 μM) on Day 4 after adipogenic stimulation. Data represent mean ± SEM; *n* = 7 per group. ^*^P < 0.05 compared to *LacZ* KD on the same treatment. ^#^*P* < 0.05 compared to the non-treated group on the same cell line.

C/EBPα and PPARγ induce their expression levels by reciprocally binding to each other’s promoter region in adipocyte differentiation *(**3**)*. Therefore, we hypothesized that rosiglitazone, a PPARγ agonist *(**17**,*  *18**)*, could restore adipocyte differentiation inhibited by *Naxd* silencing. Treatment with rosiglitazone after Day 2 of adipogenic stimuli and thereafter marginally promoted lipid accumulation with increased expression of C/EBPα in the nuclei of *LacZ* KD (Fig. 4A–D). In contrast, rosiglitazone completely restored lipid accumulation in *Naxd* KD compared to that in *LacZ* KD (Fig. 4A and B). We also found that nuclear protein levels of C/EBPα and PPARγ were substantially elevated by rosiglitazone in *Naxd* KD (Fig. 4C and D). These results suggest that NAXD maintains adipocyte differentiation presumably by regulating PPARγ transcription activity.

### Exposure to NADHX inhibits normal adipocyte differentiation in 3T3-L1 cells

**Fig. 5:**
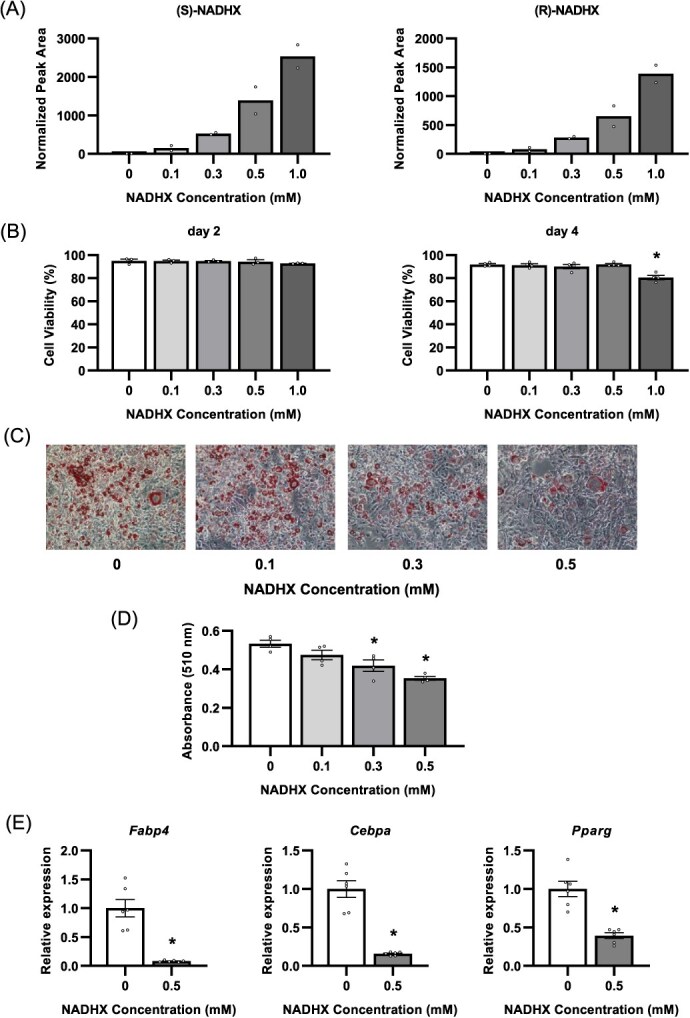
Addition of NADHX to the culture medium inhibits adipocyte differentiation of wild-type 3T3-L1 cells. (A) Quantitative analysis of amounts of NADHX in wild-type 3T3-L1 cells 1 h after exposure to the indicated concentrations of NADHX. Data represent mean ± SEM; *n* = 2 per concentration. (B) Evaluation of cell viability of wild-type 3T3-L1 cells exposed to the indicated concentrations of NADHX for the first 2 days assessed by trypan blue assay at Day 2 (left) and Day 4 (right) after induction of differentiation. Data represent mean ± SEM; *n* = 3 (Day 2) and 4 (Day 4). ^*^P < 0.05 compared to the non-treated group. Representative images of Oil red O staining (C) and corresponding quantification of Oil red O (D) of wild-type 3T3-L1 cells treated with the indicated concentrations of NADHX for the first 2 days on Day 6 after adipogenic stimulation. Scale bar, 200 μm. Data represent mean ± SEM; *n* = 4 per group. ^*^P < 0.05 compared to the non-treated group. (E) Relative quantification of *Fabp4* (left), *Cebpa* (centre) and *Pparg* (right) in wild-type 3T3-L1 cells treated with 0.5 mM NADHX for the first 2 days on Day 4 after adipogenic stimulation. Data represent mean ± SEM; *n* = 6 per group. ^*^P < 0.05 compared to the non-treated group.

We hypothesized that inhibition of adipocyte differentiation by *Naxd* silencing was attributable to excessive accumulation of NADHX. To prove this, we exposed wild-type 3T3-L1 cells to NADHX and induced adipocyte differentiation. We found that NADHX was successfully detected in cells 1 h after administration of NADHX into the culture medium, and its levels increased in a dose-dependent manner (Fig. 5A). As exposure to 1 mM of NADHX decreased cell viability assessed by trypan blue exclusion test at Day 4, but not Day 2 of adipogenic induction (Fig. 5B), we applied <0.5 mM of NADHX to examine the effects of adipocyte differentiation in the further experiments. Importantly, as observed in *Naxd* KD, exposure to NADHX substantially inhibited lipid accumulation in a dose-dependent manner after adipogenic induction (Fig. 5C and D). In addition, gene expressions of *Fabp4*, *Cebpa* and *Pparg* were reduced at Day 4 after adipogenic induction (Fig. 5E). These results suggest that NADHX inhibits adipocyte differentiation by suppressing key adipogenic transcription factors, supporting an idea that NAXD maintains normal adipogenesis of 3T3-L1 preadipocytes by inhibiting excessive accumulation of NADHX.

## Discussion

Obesity, a condition of increased adipose tissue mass, is closely associated with various metabolic dysfunctions such as diabetes, hyperlipidemia, liver steatosis and atherosclerosis *(**19**)*. Adipose tissue expansion occurs either by enlarging the size of adipocytes through storing excessive lipids or by increasing the number of adipocytes through activating adipogenesis. The well-controlled proliferation and maturation of adipocytes is crucial for maintaining healthy adipose tissue *(**20**,*  *21**)*; therefore, considerable effort has been devoted to understanding the molecular mechanisms of how transcription factors regulate the differentiation of progenitor cells into mature adipocytes. Among metabolites involved in transcriptional regulation including acetyl CoA, S-adenosylmethionine and α-ketoglutarate *(**8**,*  *22**,*  *23**)*, NAD^+^ is a crucial regulator of adipocyte differentiation, and its elevation is required to promote adipogenesis, either by activating the NAD^+^ salvage pathway *(**8**)* or by metabolic reprogramming of glycolysis and mitochondrial metabolism *(**24**)*. In this study, we first hypothesized that NAXD plays a role in adipogenesis by supporting the differentiation-induced increase in NAD^+^ by repairing hydrated NADH (NADHX), a toxic metabolite of NADH, to NADH. Here, we show that NAXD is required for adipocyte differentiation, but unexpectedly that NAXD controls the differentiation process presumably by inhibiting excessive accumulation of NADHX.

Targeted LC–MS/MS analysis revealed a time-dependent increase in NADHX levels after the induction of adipocyte differentiation, suggesting that adipogenesis enhances NADHX production and accumulation in 3T3-L1 cells. This is an unexpected finding, because there are no reports showing accumulation of NADHX in normal cells or tissues, and NADHX accumulation is thought to occur only under stress conditions. NADHX is produced either by non-enzymatic reactions (acidic pH and high temperature) or by a side reaction with GAPDH *(**11**,*  *25**)*. NADH is non-enzymatically hydrated to form NADHX at physiological temperatures at a rate of ~2% per hour *(**26**)*; however, this reaction requires non-physiological conditions such as high phosphate concentration and acidic pH *(**26**)*, excluding the possible involvement of non-enzymatic reactions in NADHX production during adipocyte differentiation. In contrast, given that adipogenesis induces metabolic reprogramming with increased GAPDH expression and subsequent activation of glycolytic flux *(**27**,*  *28**)*, GAPDH is likely to serve as the primary machinery for NADHX production. Additionally, as the activation of NAMPT in the salvage pathway of NAD^+^ leads to an increase in NAD^+^ pools in cells treated with adipogenic stimuli *(**8**)*, the expansion of the NAD^+^ pool could also stimulate NADHX accumulation through enzymatic reactions. Another important aspect of enhanced NADHX production is its effect on the cellular NAD(H) pool. Skin fibroblasts isolated from *Naxd*-deficient patients have been reported to contain NAD^+^ and NADH levels comparable to those in wild-type cells, with excessive accumulation of NADHX *(**15**)*. Consistent with this report, we observed that silencing of *Naxd* gene shows little effect on the NAD(H) pool in 3T3-L1 cells, irrespective of the differentiation status. Although this is likely due to a slow rate of NADH consumption to produce NADHX and/or a lower concentration of NADH compared to NAD^+^, our present results suggest that NAXD plays a dispensable role in the regulation of the NAD(H) pool, at least in undifferentiated and differentiating preadipocytes. It would be interesting to further investigate whether NAXD is involved in maintaining NAD(H) levels under certain stress conditions such as hypoxia and in cancerous cells, where glycolysis is constitutively activated with high GAPDH activity and thus NADH may be highly metabolized to NADHX. In contrast, our present findings showing NADHX inhibited adipocyte differentiation of wild-type 3T3-L1 cells support the idea that NAXD maintains normal adipocyte differentiation exclusively by eliminating excessive NADHX accumulation. Consistent with this idea, supplementation with NMN failed to restore adipocyte differentiation of FK866-treated, NAD^+^-depleted *Naxd* KD to the levels in *LacZ* KD treated with FK866 and NMN, although NAD^+^ levels returned comparably in FK866-treated *Naxd* KD and *LacZ* KD by NMN supplementation. Taken together, our results suggest that NAXD serves as essential machinery to maintain normal adipocyte differentiation by eliminating excessive NADHX accumulation.

PPARγ is a master regulator for adipogenesis, and a coordinated upregulation of PPARγ is a prerequisite for adipocyte differentiation initiation in 3T3-L1 cells *(**29**)*. We found that silencing of *Naxd* failed to increase nuclear protein and mRNA levels of *Pparg* with reduced *Fabp4* expression, suggesting NAXD plays a role in the regulation of adipogenesis, in part, by activating PPARγ. C/EBPβ is a pioneer transcription factor in the early phase of adipocyte differentiation and activates *Pparg* expression *(**30**,*  *31**)*. However, given that *Cebpb* expression levels and C/EBPβ binding to the promoter region of *Pparg* were comparable between control and *Naxd* KD, C/EBPβ-mediated induction of *Pparg* was not impaired by the suppression of *Naxd*, suggesting a dispensable role of C/EBPβ in the NAXD-mediated regulation of adipogenesis. C/EBPα and PPARγ reciprocally upregulate each other’s transcription during adipogenesis and simultaneously bind to the same region in the *Pparg2* gene locus *(**3**,*  *32**,*  *33**)*. Therefore, our results do not exclude the inhibitory effects of *Naxd* silencing on C/EBPα, as the protein and mRNA levels of *Cebpa* were also suppressed in *Naxd* KD. However, treatment with rosiglitazone restored adipocyte differentiation in *Naxd* KD comparably to *LacZ* KD with increased PPARγ and C/EBPα expression, supporting the idea that *Naxd* deficiency preferentially shows an inhibitory effect on the transcriptional activity of PPARγ, resulting in impaired adipocyte differentiation. The transcriptional activity of PPARγ has also been reported to be regulated by post-translational modifications such as phosphorylation *(**34**)*, SUMOylation *(**35**)* and acetylation *(**36**)*. Rosiglitazone stimulates deacetylation and subsequent activation of PPARγ by promoting binding to sirtuin 1 (SIRT1), a NAD^+^-dependent deacetylase, leading to the browning of white adipose tissue *in vivo (**36**)*. Considering the structural similarity between NAD^+^ and NADHX, the excessive accumulation of NADHX in early adipocyte differentiation of *Naxd* KD, and inhibitory effects of NADHX on *Pparg* induction and subsequent adipocyte differentiation, it would be interesting to address whether NAXD supports normal adipocyte differentiation by inhibiting excessive accumulation of NADHX and subsequently regulating SIRT1-mediated PPARγ transcriptional activity in early adipocyte differentiation. Another hypothesis is that NADHX controls the epigenetic regulation and subsequently inhibits expression of *Pparg* and *Cebpa* genes. NADHX has been reported to inhibit 3-phosphoglycerate dehydrogenase activity with decreased *de novo* serine synthesis *(**14**)*. Serine is a critical metabolite for the supply of S-adenosylmethionine (SAM), a methyl donor for various proteins including histones and DNA *(**37**)*. H3K4me1/2 by MLL4 (KMT2D) has been reported to play a central role in enhancer activation of *Pparg* and *Cebpa* gene in adipocyte differentiation *(**38**)*. Based on these reports, it is also plausible that NADHX abrogates induction of *Pparg* and *Cebpa* gene expressions by inhibiting *de novo* serine synthesis and consequently decreasing histone modifications marked by H3K4me1/2, resulting in inhibition of adipocyte differentiation, although further investigation is needed to prove this hypothesis.

In conclusion, we demonstrated that suppression of *Naxd* inhibits normal adipocyte differentiation presumably by stimulating excessive NADHX accumulation and subsequently attenuating *Pparg* induction in preadipocyte 3T3-L1 cells. Furthermore, adipocyte differentiation stimulates NADHX formation, and NAXD successfully suppresses the excessive accumulation of the toxic metabolite NADHX with little effect on the NAD^+^ pool. The present study deepens our knowledge of the importance of the NAXD-mediated repair mechanisms of NADHX in NAD^+^ metabolism and highlights the necessity to further investigate the possible involvement of NADHX in various NAD^+^-associated diseases such as cancer, ageing and metabolic diseases.

## Supplementary Material

Web_Material_mvaf015
