# Supplementary material for: Suppression of ATP-dependent (S)-NAD(P)H-hydrate dehydratase expression inhibits adipocyte differentiation of 3T3-L1 preadipocytes by increasing excessive accumulation of NADHX
Source: J Biochem. 2025 Mar 21;177(6):403–14. doi: 10.1093/jb/mvaf015 (PMC12136578; doi:10.1093/jb/mvaf015)
Supplement: Web_Material_mvaf015 [file web_material_mvaf015.docx]

Supplemental Information for

Suppression of ATP-dependent (S)-NAD(P)H-hydrate dehydratase expression inhibits adipocyte differentiation of 3T3-L1 preadipocytes by increasing excessive accumulation of NADHX

Kazuki Nakajima ^1^, Kodai Takahashi^1^, Masako Tanaka^2^, Mina Kawashima^1^, Koshi Machida^3^, Yoichi Nakao^3^, Keiyo Takubo^4,5^, Nobuhito Goda^1^*

Corresponding author and Lead Contact:

Nobuhito Goda, M.D., Ph.D.

E-mail: [goda@waseda.jp](mailto:goda@waseda.jp)

^1^Department of Life Science and Medical Bioscience, Graduate School of Advanced Science and Engineering, Waseda University, Tokyo, 162-8480, Japan

^2^Department of Biomedical Sciences, School of Biological and Environmental Sciences, Kwansei Gakuin University, Hyogo, 669-1330, Japan

^3^Department of Chemistry and Biochemistry, Graduate School of Advanced Science and Engineering, Waseda University, Tokyo, 169-8555, Japan

^4^Department of Cell Fate Biology and Stem Cell Medicine, Tohoku University Graduate School of Medicine, Sendai, 980-8575, Japan

^5^Department of Stem Cell Biology, Research Institute, National Center for Global Health and Medicine, Tokyo, 162-8655, Japan

Supplemental Figures: page 2

Supplemental Tables: page 3

Supplemental Figures

(A)


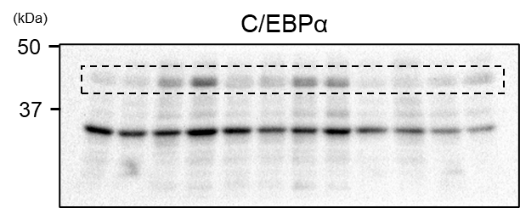

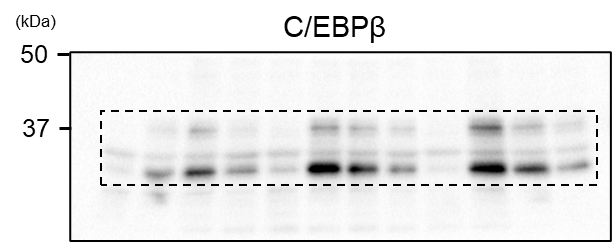


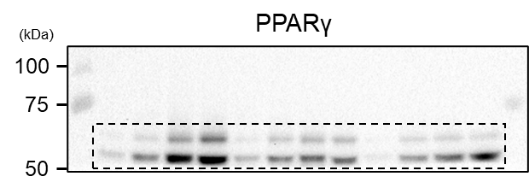

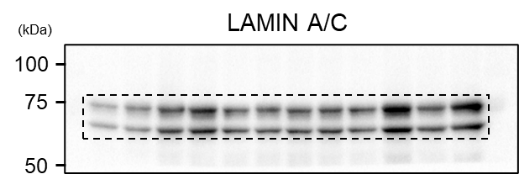


(B)


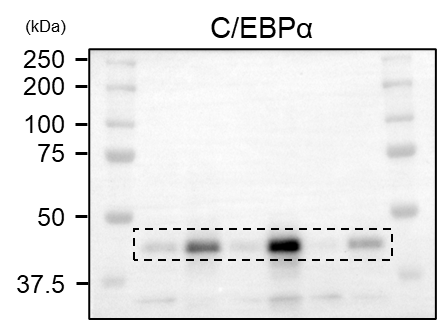

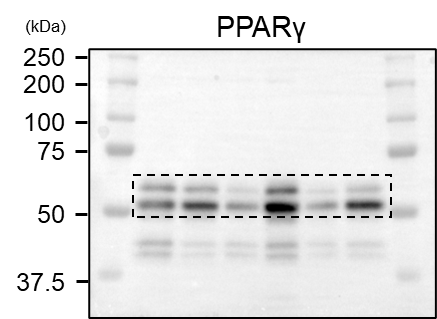

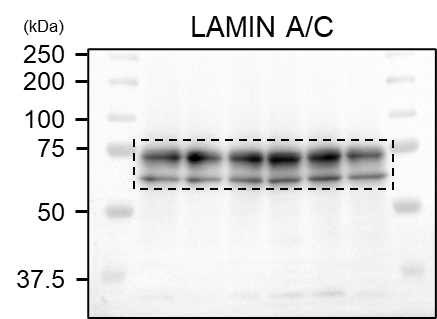


Supplemental Figure S1.

Uncropped Western blotting membrane diagrams. (A) and (B) correspond to the membrane diagrams in Figure 3. (A) and Figure 4. (C), respectively.


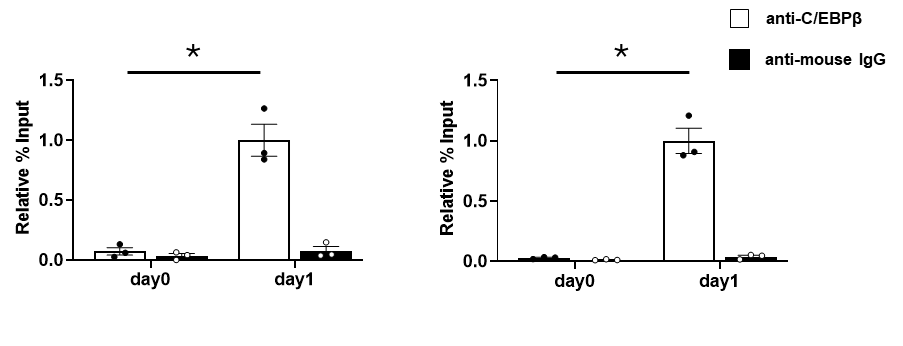


Supplemental Figure S2.

ChIP-qPCR analysis of C/EBPβ at the promoter of *Cebpa* (left) and *Pparg* (right) gene in *Naxd*-deficient 3T3-L1 cells after adipogenic stimulation. Data represent mean ± SEM; n = 3 per group. *: *p* < 0.05

Supplemental Tables

Supplemental Table S1. sh Sequence for knockdown cells establishment

| Target gene | sh sequence (5’ – 3’) | Loop sequence (5’-3’) |
| --- | --- | --- |
| *LacZ* | GCAGTTATCTGGAAGTACA | TTCAAGAGA |
| *Naxd #1* | ACCACTACCGACATGATCACAGAG |  |
| *Naxd #2* | AGCAGGACCTGATCTCCAAGG |  |

Supplemental Table S2. Primer sequence for expression analysis

| Target gene | Forward (5’-3’) | Reverse (5’-3’) |
| --- | --- | --- |
| *18S rRNA* | GCTCGCTCCTCTCCTACTTG | CCCGTCGGCATGTATTAGCT |
| *Naxd* | ACCACAAGGCCATTCTCACC | TGGAGCCCTTGAGATCGTTG |
| *Cebpa* | AGCTGAGTTGTGAGTTAGCCATGT | ACCCCACAAAGCCCAGAAA |
| *Fabp4* | GACGACAGGAAGGTGAAGAGC | CACATTCCACCACCAGCTTGTC |
| *Pparg* | GGGATGTCTCACAATGCCATC | TGGGTTCAGCTGGTCGATATC |

Supplemental Table S3. Detail of antibodies

| antibody | Supplier | Catalog No. |
| --- | --- | --- |
| anti-C/EBPα | Cell Signaling Technology | #8178 |
| anti-C/EBPβ | Santa Cruz | sc-7962 |
| anti-LAMIN A/C | Santa Cruz | sc-376248 |
| anti-mouse IgG HRP | Cell Signaling Technology | #7076 |
| anti-PPARγ | Santa Cruz | sc-7273 |
| anti-rabbit IgG HRP | Cytiva | NA930 |

Supplemental Table S4. Primer sequence for ChIP-PCR

| Target Region | Forward (5’-3’) | Reverse (5’-3’) |
| --- | --- | --- |
| *Cebpa* promoter | TTAAAGGAGGGGCGCCTAAC | AGTGCTAGTGGAGAGAGATCG |
| *Pparg* promoter | CTGTACAGTTCACGCCCCTC | CTGTCTGCTGCTTTGGCAAG |

Supplemental Table S5. Solvent species and gradients for mass spectrometry

| Items | Settings |
| --- | --- |
| Solvent A | 50mM Ammonium acetate |
| Solvent B | Acetonitrile |
| Gradient (%B) | 0 min: 0  5 min: 0  23 min: 6  28 min: 100  33 min: 100  34 min: 0  48 min: 0 |

Supplemental Table S6. Mass value and retention time of target metabolite

| Target Metabolite | MS number (*m*/*z*) | | Retention time (min) |
| --- | --- | --- | --- |
|  | precursor ion | product ion |  |
| NAD^+^ | 540.10 | 328.200 | 12.7 |
| NADH | 664.11 | 397.150 | 15.38 |
| S-NADHX | 682.12 | 346.051 | 9.58 |
| R-NADHX | 682.12 | 346.051 | 13.96 |
| Cyclic NADHX | 664.11 | 397.150 | 17.71 |
